# Neonatal thyroid-stimulating hormone concentration and psychomotor development at preschool age

**DOI:** 10.1136/archdischild-2015-310006

**Published:** 2016-07-08

**Authors:** Caroline Trumpff, Jean De Schepper, Johan Vanderfaeillie, Nathalie Vercruysse, Herman Van Oyen, Rodrigo Moreno-Reyes, Jean Tafforeau, Stefanie Vandevijvere

**Affiliations:** 1 Unit of Public Health and Surveillance, Scientific Institute of Public Health, Brussels, Belgium; 2 Department of Paediatric Endocrinology, UZ Brussel, Vrije Universiteit Brussel, Brussels, Belgium; 3 Faculty of Psychology and Educational Sciences, Vrije Universiteit Brussel, Brussels, Belgium; 4 Faculty of Psychology and Educational Sciences, Université Libre de Bruxelles, Brussels, Belgium; 5 Department of Nuclear Medicine, Erasme Hospital, Université Libre de Bruxelles, Brussels, Belgium

**Keywords:** psychomotor development, thyroid-stimulating hormone, preschool children, iodine deficiency, pregnancy

## Abstract

**Objective:**

Thyroid hormones are essential for normal brain development. The aim of this study is to assess if high concentration of thyroid stimulating hormone (TSH) that is below the clinical threshold (5–15 mIU/L) at neonatal screening is linked to psychomotor development impairments in the offspring at preschool age.

**Design:**

A total of 284 Belgian preschool children 4–6 years old and their mothers were included in the study. The children were randomly selected from the total list of neonates screened in 2008, 2009 and 2010 by the Brussels newborn screening centre. The sampling was stratified by gender and TSH range (0.45–15 mIU/L). Infants with congenital hypothyroidism (>15 mIU/L), low birth weight and/or prematurity were excluded. Psychomotor development was assessed using the Charlop-Atwell scale of motor coordination. The iodine status of children was determined using median urinary iodine concentration. Socioeconomic, parental and child potential confounding factors were measured through a self-administered questionnaire.

**Results:**

TSH level was not significantly associated with total motor score (average change in z-score per unit increase in TSH is 0.02 (−0.03, 0.07), p=0.351), objective motor score (p=0.794) and subjective motor score (p=0.124). No significant associations were found using multivariate regression model to control confounding factors.

**Conclusions:**

Mild thyroid dysfunction in the newborn—reflected by an elevation of TSH that is below the clinical threshold (5–15 mIU/L)—was not associated with impaired psychomotor development at preschool age.

**What is already known on this topic?**

- Thyroid hormones are essential for proper brain development.
- A severe lack of thyroid hormones can lead to permanent intellectual disability and psychomotor deficits.
- Little is known about the long-term consequences of mild thyroid dysfunction at birth on later psychomotor development.

**What this study adds?**

- No significant association was found between neonatal TSH concentration and psychomotor scores at preschool age.
- No impact of mild thyroid dysfunction at birth was found on psychomotor development of preschool children.

## Introduction

Thyroid hormones, thyroxin (T4) and tri-iodothyronine (T3) are essential for fetal growth and optimal brain development in utero and after birth.^1^ During pregnancy, a sufficient production and transfer of thyroid hormones from the mother to the fetus is needed for healthy fetal brain development. Indeed thyroid hormones are involved in neural migration, neural differentiation, myelinisation, synaptogenesis and neurotransmission.^2^

Adequate iodine intake is essential for the production of thyroid hormones. Severe iodine deficiency can lead to congenital hypothyroidism in the neonate, a condition characterised by severe intellectual disability, dwarfism, deaf-mutism and spasticity.^3^ At mild to moderate levels, iodine deficiency can lead to subclinical thyroid dysfunction in the mother and the fetus and alter children's neurocognitive development.^4^ Epidemiological studies have shown that maternal hypothyroxinaemia or low maternal urinary excretion appearing during pregnancy could lead to subclinical impairments in verbal and non-verbal skills, perceptual and manipulative abilities, executive functioning skills and memory abilities.^4^

Besides intellectual disability, motor deficits are commonly observed in children with congenital hypothyroidism with alteration in gross and fine motor abilities, motor coordination and static balance.^5–7^ At the subclinical level, maternal hypothyroxinaemia has been found to be associated with altered gross motor abilities.^8^ ^9^ Results from animal studies have shown that maternal thyroid hormones deficiency can alter cerebellum development and function and lead to motor coordination impairments in the offspring.^10–12^

Iodine deficiency is still present at mild levels in several European countries, including Belgium, and can lead to subclinical thyroid dysfunction in both the mother and the offspring.^13^ ^14^ Little is known about the potential harmful effects of mild thyroid dysfunction at birth on later neurodevelopment of children. This is a public health concern and warrants further study in view of the sparse evidence available.

Thyroid hormone level is dependent on iodine nutrition when thyroid disease is excluded.^15^ The neonatal thyroid is very sensitive to variations of maternal iodine intake. This is the reason why thyroid stimulating hormone (TSH) concentration measured 3–5 days after birth has been proposed as an indicator of population iodine status using the percentage of newborns with a neonatal TSH above 5 mIU/L.^15–19^

To our knowledge, only two studies have investigated the association between high (>5 mIU/L) neonatal TSH as a marker of thyroid dysfunction and psychomotor development in young children.^20^ ^21^

The aim of the present study is to assess the effect of mild thyroid dysfunction at birth—potentially secondary to maternal iodine deficiency—on psychomotor development at preschool age by investigating the association between neonatal TSH concentrations and psychomotor scores at preschool age.

### Method

This study included 284 Belgian preschool children aged 4–6 years with a neonatal TSH concentration in the range 0.45–15 mIU/L (micro international U/L) from the PsychoTSH cohort study,^22^ ^23^ which aimed to assess the association between neonatal TSH and cognitive, psychomotor and psychosocial development. The children were selected from the total list of newborns screened by the Brussels Newborn Screening Centre for Metabolic Disorders (Laboratoire de Pédiatrie, Université Libre de Bruxelles, Brussels) in 2008 (n=29 013), 2009 (n=29 602) and 2010 (n=30 126). The sampling was stratified by gender and TSH interval (0.45–1 mIU/L, 1–2 mIU/L, 2–3 mIU/L, 3–4 mIU/L, 4–5 mIU/L, 5–6 mIU/L, 6–7 mIU/L, 7–8 mIU/L, 8–9 mIU/L and 9–15 mIU/L). The sample size has been calculated for the relationship between TSH and cognitive score and is described elsewhere.^22^ ^23^ Infants with congenital hypothyroidism (TSH concentration >15 mIU/L), prematurity (<37 weeks), low birth weight (<2500 g) and neurological disease were excluded from the sample.

TSH concentration was measured in dried blood spots on filter paper collected 3–5 days after birth using a commercial time-resolved fluoroimmunoassay (Autodelfia method).^24^ TSH values were analysed twice at 50 different TSH values in the range of 0–15 mIU/L to assess precision by determining the coefficient of variation. The interday and intraday coefficient of variation was below 20% in the range 0.9–15 mIU/L. For values <0.9 mIU/L, a weighted value of 0.45 mIU/L was given to include these data in statistical analysis.

Psychomotor development of children was assessed with the French version of the Charlop-Atwell Scale of Motor Coordination.^25^ ^26^ This is an individually administered test evaluating gross motor coordination abilities of children aged 3½–6 years using six subtests. The French version of the test has good interobserver reliability, and internal and external validity.^26^ The scores comprise an objective subtest rating based on the accuracy of the performance and a subjective rating based on the quality of the performance. The test was performed at home with a psychologist who was blinded to the neonatal TSH value of the children.

A sample of urine was collected from the child in order to assess iodine status of the studied population. Samples were frozen at −80°C and urinary iodine excretion was measured by a modification of the Sandell-Kolthoff reaction with spectrophotometric detection^27^ at the Nuclear Medicine Department, Erasmus Hospital (Université Libre de Bruxelles, Brussels). During the home visit, actual body measurement of the child was taken. The body weight was determined using a SECA 815 or SECA 804 weight scale, the body height using a transportable SECA 214 stadiometer and the head circumference using a SECA 212 flexible measuring tape (seca GmbH. Co. kg, Germany).

Descriptive data, data on effect modifiers and covariates were collected from the screening centre (date of birth, date of blood sampling, pregnancy duration and body weight at birth), the health booklet of the child (body length at birth, head circumference at birth and Apgar score) and by a self-reported questionnaire filled in by the mothers.

The following information was assessed by the self-report questionnaire: prepregnancy body mass index and weight gain of the mother during pregnancy, thyroid disease, diabetes, drug intake and nutritional supplements intake during pregnancy, alcohol consumption, smoking habits, maternal age at birth, reproductive history, parity, gravidity, type of delivery, health condition of the newborn at birth, perinatal asphyxia, child's negative life events, child's chronic disease, child's lifetime nutritional supplement intake, maternal social support, marital discord and parent-child interactions, and maternal mental health. Maternal mental health was assessed using the General Health Questionnaire-12 items and vitality scale questionnaire of the Short Form Health Survey-36.

Statistical data analysis was performed using SAS statistical software V.9.3 (SAS Institute Cary, North Carolina, USA) for univariate analysis and Stata V.13 (StataCorp, College Station, Texas, USA) for multivariable analysis. Tests were two-sided and p values <0.05 was considered as statistically significant. Urinary iodine concentration and TSH values were expressed as median and IQR. Psychomotor scores were presented as z-scores and were standardised using the mean and SD by age group of the French population of reference.^26^ These scores were presented as mean and SD. Neonatal TSH values were analysed as ranks using 1 mIU/L interval or were classified in two groups: below 5 mIU/L and higher than 5 mIU/L.

Univariate associations between TSH concentration at birth and psychomotor scores were assessed using Pearson's correlation. To analyse the association between psychomotor scores and studied parameters, Student's t-test, analysis of variance (ANOVA) with Bonferroni correction and simple linear regression tests were performed.

Multivariable linear regression models were used to define the predictors of variation of psychomotor scores in children. All variables associated with psychomotor scores with a test p<0.20 were included in a stepwise backward selection procedure with a probability of entry of 0.10 and probability of exit of 0.15. The variables kept by this selection procedure were inserted in the final multiple linear regression model (except for the TSH value which was kept even without being selected). The normality of the distribution of residuals of each multivariable linear model was tested using a normal plot of residuals. Linearity and homoscedasticity of residuals were checked by analysing the plot of standardised residuals. Colinearity between predictors was tested using the test of variance inflation factor (VIF). Total VIF and individual VIF for each parameter in the model were around 1. Variables highly correlated with each other were not included together in the model. In addition, a second multivariable model was tested using a multiple imputation strategy to fill in missing values (4%) using the “mi” command in Stata V.13 (StataCorp, College Station, Texas, USA) with five imputations.

## Results

The characteristics of the preschoolers are shown in table 1. The median TSH level was 3.7 mIU/L (1.81–5.9 (IQR), 0.45–13.9 (min–max)). The median urinary iodine concentration was 137 µg/L (88.2–233.6 (IQR)) indicating iodine sufficiency of the study population.

**Table 1:** Descriptive and demographic characteristics of the studied population according to gender

(N=284)	Total		Male		Female	
	N	Per cent	N	Per cent	N	Per cent
*TSH level (mIU/L)*						
1	41	14	22	8	19	7
2	35	12	21	7	14	5
3	35	12	20	7	15	5
4	35	12	16	6	19	7
5	35	12	20	7	15	5
6	36	13	19	7	17	6
7	33	12	20	7	13	5
8	12	4	9	3	3	1
9	13	5	6	2	7	2
10–15	9	3	5	2	4	1
*Age at testing*						
4	211	75	118	42	93	33
5	65	23	36	13	29	10
6	7	2	4	1	3	1
*Maternity hospital*						
Brussels	190	67	117	41	73	26
Wallonia	93	33	41	14	52	18
Flanders	1	0	–	–	1	0
*Home at examination*						
Brussels	103	37	57	20	46	16
Wallonia	131	47	70	25	61	22
Flanders	45	16	28	10	17	6
*Children ethnicity*						
Europe (Caucasian)	226	85	129	48	97	36
Asian	3	1	2	1	1	0
Sub-Saharan Africa	13	5	5	2	8	3
North Africa	25	9	14	5	11	4
*Mother ethnicity*						
Europe (Caucasian)	226	83	130	48	96	35
Asia	5	2	2	1	3	1
Sub-Saharan Africa	15	6	6	2	9	3
North Africa	26	10	15	6	11	4
*Father ethnicity*						
Europe (Caucasian)	228	84	129	48	99	37
Asia	2	1	–	–	2	1
Sub-Saharan Africa	14	5	5	2	9	3
North Africa	26	10	14	5	12	4

PsychoTSH study, Belgium, 2008–2014.

N, number of subjects; TSH, thyroid stimulating hormone.

The mean total motor score was −0.03 (1.1 (SD), −3.5–2.11 (min–max)), the mean objective motor score was 0.2 (1.1 (SD), −3.7–1.7 (min–max)), the mean subjective motor score was 0.28 (1.1 (SD), −2.9–2.5 (min–max)).

Univariate associations between psychomotor scores at preschool age and maternal, socioeconomic characteristics and markers of iodine status were tested. The results are shown in tables 2 and 3.

**Table 2:** Association of psychomotor scores at preschool age with infant, maternal, socioeconomic characteristics and markers of iodine status: categorical variables

	Total		Total motor scale			Objective motor scale			Subjective motor scale		
	N	Per cent	Mean	SD	p Value	Mean	SD	p Value	Mean	SD	p Value
*Children characteristics*											
Gender					**<0.0001**			**0.0004**			**<0.0001**
Male	158	54.60	−0.29	1.19		−0.43	1.17		0.02	1.09	
Female	126	45.40	0.30	0.94		0.03	0.92		0.61	0.93	
*Socioeconomic characteristics and maternal characteristics*											
Monthly income					0.112			0.451			0.183
<€2000	33	12.67	−0.51	1.17		0.15	1.12		−0.27	1.23	
≥€2000	240	87.33	−0.19	1.07		0.30	1.05		0.01	1.10	
Mother education level					0.262			0.939			0.513
No/primary	10	3.28	−0.46	1.28		0.18	1.44		−0.20	1.39	
Lower high school	20	7.21	−0.58	1.23		0.17	1.30		−0.33	1.38	
Upper high school	44	16.39	−0.31	1.14		0.28	1.05		−0.07	1.14	
University or higher	201	73.11	−0.15	1.03		0.30	1.03		0.03	1.07	
Delivery					0.066			0.107			0.071
Normal	213	75.73	−0.21	1.04		0.26	1.03		−0.03	1.07	
Caesarean	39	13.92	0.00	1.22		0.59	1.22		0.26	1.30	
With vacuum	27	10.03	−0.63	1.24		0.06	1.10		−0.39	1.27	
Parity—first child					**0.010**			**0.004**			0.116
Yes	121	43.37	−0.23	1.17		−0.44	1.13		0.17	1.08	
No	158	56.63	0.13	1.08		−0.06	1.04		0.37	1.05	
Smoking during pregnancy					0.405			0.118			0.234
≥10 cigarettes/day	6	2.11	−0.60	1.19		−0.39	1.58		−0.57	1.43	
<10 cigarettes/day or non-smoking	278	97.89	−0.22	1.09		0.30	1.05		−0.02	1.12	
Alcohol during pregnancy					0.172			0.978			0.392
Non-consumer	188	68.08	−0.28	1.11		0.29	1.10		−0.06	1.17	
Still consuming	89	31.92	−0.09	1.04		0.29	1.01		0.06	1.04	
*Markers of iodine status*											
Neonatal TSH level					0.517			0.141			0.270
<5	181	64.42	−0.26	1.02		0.21	0.98		−0.09	1.03	
>5	103	35.58	−0.17	1.21		0.40	1.19		0.07	1.28	
Vitamins during pregnancy					0.705			0.527			0.898
Containing iodine	48	43.59	0.06	1.17		−0.07	1.04		0.25	1.19	
No vitamins	57	56.41	−0.01	0.97		−0.19	0.97		0.27	0.93	
Urinary iodine concentration, lg/L					0.544			0.398			0.648
<100	72	30.37	0.08	1.04		−0.13	1.01		0.36	0.98	
100–149	63	24.81	−0.04	1.12		−0.16	1.13		0.16	1.06	
150–294	55	22.22	0.05	1.07		−0.16	1.03		0.35	1.02	
≥250	53	22.59	−0.20	1.27		−0.43	1.15		0.21	1.25	
Household salt					0.097			0.181			0.135
Iodised salt	84	34.53	−0.10	1.16		−0.25	1.10		0.17	1.11	
Non-iodised salt	154	61.15	0.08	1.08		−0.14	1.06		0.38	1.04	
No salt	10	4.32	−0.62	0.71		−0.76	0.68		−0.18	0.91	

PsychoTSH study, Belgium, 2008–2014.

Significant associations (p values <0.05) are marked in bold. p Value from Student's t-test or analysis of variance (ANOVA).

N, number of subjects; TSH, thyroid stimulating hormone.

**Table 3:** Association of psychomotor scores at preschool age with infant, maternal, socioeconomic characteristics and markers of iodine status: continuous variables

				Total motor scale			Objective motor scale			Subjective motor scale		
	N	Mean/median*	SD/IQR*	Linear regression								
				b	95% CI	p Value	b	95% CI	p Value	b	95% CI	p Value
TSH (mUI/L)	284	3.7*	1.8, 5.9*	0.02	−0.03 to 0.07	0.351	0.01	−0.04 to 0.05	0.794	0.04	−0.01 to 0.08	0.124
Term pregnancy (week)	278	39.3	1.6	−0.05	−0.13 to 0.03	0.215	−0.05	−0.13 to 0.03	0.215	−0.05	−0.12 to 0.03	0.226
Birth weight (g)	283	3406.7	428.3	0.00	−0.00 to 0.00	0.652	0.00	−0.00 to 0.00	0.498	0.00	−0.00 to 0.00	0.817
Birth length (cm)	279	50.0	3.2	−0.02	−0.06 to 0.02	0.379	−0.02	−0.05 to 0.02	0.388	−0.02	−0.05 to 0.02	0.434
Birth HC (cm)	245	34.6	3.3	−0.01	−0.05 to 0.02	0.528	−0.01	−0.05 to 0.03	0.708	−0.02	−0.05 to 0.02	0.455
Actual age (year)	283	4.7	0.5	−0.45	−0.72 to −0.17	**0.002**	−0.47	−0.73 to −0.20	**0.001**	−0.29	−0.54 to −0.03	**0.030**
Actual weight (kg)	280	18.6	3.0	−0.05	−0.10 to−0.01	**0.013**	−0.05	−0.09 to −0.01	**0.011**	−0.04	−0.08 to 0.00	0.051
Actual height (cm)	281	107.9	8.1	−0.02	−0.04 to−0.00	**0.010**	−0.02	−0.03 to −0.00	**0.027**	−0.02	−0.04 to −0.01	**0.006**
Actual HC (cm)	280	51.3	3.3	−0.03	−0.07 to 0.01	0.142	−0.02	−0.06 to 0.01	0.238	−0.03	−0.07 to 0.00	0.098
Mother age (years)	280	36.5	5.1	0.00	−0.02 to 0.03	0.817	0.00	−0.02 to 0.03	0.802	0.00	−0.02 to 0.03	0.802
Mother weight (kg)	277	65.1	12.0	0.00	−0.02 to 0.00	0.381	0.00	−0.01 to 0.00	0.384	0.00	−0.01 to 0.01	0.452
Mother height (cm)	275	165.8	6.2	0.00	−0.02 to 0.02	0.951	0.01	−0.01 to 0.03	0.621	−0.01	−0.03 to 0.01	0.475
Weight gain during pregnancy (kg)	261	13.3	5.6	0.00	−0.02 to 0.02	0.969	0.00	−0.02 to 0.03	0.868	0.00	−0.02 to 0.02	0.954
Nb of cigarettes during pregnancy	17	5*	5.0, 10.0*	−0.08	−0.14 to −0.01	**0.020**	−0.07	−0.13 to 0.00	0.056	−0.09	−0.15 to −0.02	**0.014**
Nb of people in the household	274	3*	3.0, 4.0*	0.14	0.01 to 0.27	**0.029**	0.13	0.00 to 0.25	**0.049**	0.12	−0.00 to 0.24	0.051

*Median and IQR are presented. Significant associations (p values <0.05) are marked in bold.

PsychoTSH study, Belgium, 2008–2014.

p value from univariate linear regression. HC, head circumference; N, number of subjects; Nb, number; TSH, thyroid stimulating hormone.

No significant association was found between psychomotor scores and the following factors: number of previous miscarriage, Apgar score at 5 min, health problems at birth, neonatal hospital attendance, breast feeding, child food allergy, child dietary supplement intake, child's negative life events, previous cognitive assessment, school attendance, child custody, child's fish and milk consumption, rural or urban residency, type of delivery, Graves' disease or Hashimoto's thyroiditis during pregnancy, hypothyroidism during pregnancy, diabetes during pregnancy, mother's social support, mother's score of psychological distress and mother's vitality index score (data not shown).

**Figure 1:**
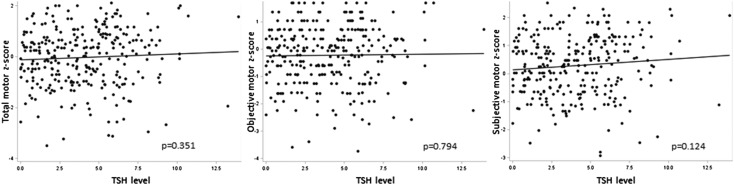
Scatter plot of the association between thyroid stimulating hormone (TSH) concentration at birth and motor z-score at preschool age.

No differences in scores were found in children with TSH levels lower than 5 mIU/L compared withTSH levels higher than 5 mIU/L. In univariate linear analysis, TSH level was not significantly associated with total motor score (average change in z-score per unit increase in TSH is 0.02 (−0.03, 0.07), p=0.351), objective motor score (average change in z-score per unit increase in TSH is 0.01 (−0.04, 0.05), p=0.794) and subjective motor score (average change in z-score per unit increase in TSH is 0.04 (−0.01, 0.08), p=0.124) (see table 3 and figure 1). No significant associations were found between TSH levels and psychomotor scores in multiple linear regression analyses with correction of covariates (see table 4). No significant associations were found either when multivariable analyses were performed using multiple imputations to replace missing values (see table 4) or when stratified for day of collection and for year of birth (data not shown).

**Table 4:** Multiple linear regressions of factors explaining variation in psychomotor scores at preschool age

Model 1 (analysis restricted to complete data)						
	Total motor scale		Objective motor scale		Subjective motor scale	
	R^2^=13% (N=275)	p Value	R^2^=13% (N=272)	p Value	R^2^=10% (N=283)	p Value
	b (95% CI)		b (95% CI)		b (95% CI)	
TSH (mIU/L)	0.04 (−0.01 to 0.09)	0.103	0.02 (−0.02 to 0.07)	0.255	0.03 (−0.01 to 0.08)	0.107
Gender		**<0**.**001**		**<0**.**001**		**<0**.**001**
Male	Ref		Ref		Ref	
Female	0.61 (0.36 to 0.87)		0.46 (0.21 to 0.71)		0.59 (0.34 to 0.83)	
Age at testing (year)	−0.41 (−0.66 to −0.15)	**0**.**002**	−0.33 (−0.59 to −0.07)	**0**.**013**	−0.27 (−0.51 to −0.04)	**0**.**022**
Parity	−0.17 (−0.31 to −0.03)	**0**.**016**	−0.23 (−0.36 to −0.91)	**0**.**001**	*	
Height at testing (cm)	*		−0.01 (−0.03 to 0.00)	0.055	*	
Model 2 (with multiple imputation of missing values)						
	Total motor scale		Objective motor scale		Subjective motor scale	
	N=284	p Value	N=284	p Value	N=284	p Value
	b (95% CI)		b (95% CI)		b (95% CI)	
TSH (mIU/L)	0.03 (−0.02 to 0.08)	0.235	0.02 (−0.03 to 0.06)	0.490	0.04 (0.01 to 0.10)	0.108
Gender		**<0**.**001**		**<0**.**001**		**<0**.**001**
Male	Ref		Ref		Ref	
Female	0.58 (0.33 to –0.83)		0.44 (0.19 to 0.68)		0.59 (0.35 to 0.82)	
Age at testing (year)	−0.41 (−0.66 to −0.16)	**0**.**001**	−0.35 (−0.60 to −0.10)	**0**.**007**	−0.28 (−0.51 to −0.4)	**0**.**022**
Parity	−0.17 (−0.31, to −0.03)	**0**.**018**	−0.21 (−0.35 to −0.07)	**0**.**003**	*	
Height at testing (cm)	*		−0.01 (−0.03 to 0.01)	0.077	*	

PsychoTSH study, Belgium, 2008–2014.

Significant associations (p values <0.05) are marked in bold.

*Variable not included in the multivariable model.

N, number of subjects; TSH, thyroid stimulating hormone.

Table 4 shows the results of multiple linear regressions assessing parameters explaining variation of psychomotor scores. Female gender was associated with higher psychomotor scores (total score, p<0.001, 95% CI 0.36 to 0.87, objective score, p<0.001, 95% CI 0.21 to 0.71, subjective score p<0.001, 95% CI 0.34 to 0.83). A negative association was found between psychomotor scores and age at testing (total score, p=0.002, 95% CI −0.66 to −0.15, objective score, p=0.013, 95% CI −0.59 to −0.07, subjective score=0.022, 95% CI −0.51 to −0.04) and number of previous births (total score, p=0.016, 95% CI −0.31 to −0.03, objective score, p=0.001, 95% CI −0.36 to −0.91).

## Discussion

In this study, we aimed to assess if elevation of TSH concentration measured 3–5 days after birth—used as a marker of mild thyroid dysfunction potentially due to maternal iodine deficiency during late pregnancy—is associated with lower psychomotor scores at preschool age. We found no association between TSH levels and psychomotor scores both in univariate and multivariable analyses.

To our knowledge, only two studies have investigated psychomotor performance of children born with an elevated TSH and those had several limitations. In Italy, a retrospective cohort study was conducted in a group of 102 infants born between 26 weeks and 32 weeks. They found that preterm newborns with a neonatal TSH value above 4.3 mIU/L had a suboptimal motor outcome at 18 months.^21^ As preterm birth is known to increase TSH levels and to be related to suboptimal motor outcome^22^ the results of this study are not applicable to children born at term. The second study is an Iranian research which assessed the effect of transient neonatal hyperthyrotropinaemia (TNH) on psychomotor performance with Bender-Gestalt test on a sample of children 9 years of age.^20^ They evaluated 18 children with TNH in comparison with 19 children without thyroid problems. They found no significant difference in psychomotor scores between the two groups. In addition to its very small sample size and to the lack of control of covariates, the results of this study are not comparable to those of the present study because children had much higher TSH values (23.4±8.3) than those included in the present study.

Several studies have investigated the effect of maternal hypothyroxinaemia during pregnancy on psychomotor development, most of them showed subtle motor impairments when hypothyroxinaemia appears during pregnancy.^4^ However, two studies found no association between hypothyroxinaemia during pregnancy and motor scores. One of them was a Spanish study which investigated motor development of McCarthy Scales of Children's Abilities of 147 children aged between 38 months and 60 months.^28^ The other investigated motor development in a population-based cohort of 1761 children with Bayley Scales of Infant Development at 2 years of age.^29^ One animal study assessing the impact of the severity of iodine deficiency on cerebellum development found no impact of mild iodine deficiency exposure on the cerebellum Purkinje cells.^30^

The present study has assessed psychomotor outcomes of children born at term with elevated TSH at birth with the exclusion of children of low birth weight and infants with congenital hypothyroidism (>15 mIU/L). In this study the sample was stratified by TSH level and by gender to ensure that the whole range of TSH values are included. In addition, psychomotor assessment of the children was done by psychologists blinded about the TSH value of the children. The assessment was performed at preschool age, reducing the time between TSH testing and psychomotor testing. For a sample size of 284, with an α error of 0.05 and a power of 0.95, the minimum score difference that we were able to detect is a difference of 3.12 points. This seems reasonable to detect children's motor development difference at the population level.

Elevated TSH concentration at birth has been considered in this study as a potential indicator of maternal iodine deficiency during late pregnancy. However, several other factors may affect TSH concentration such as maternal disease, maternal drugs intake, type of delivery and birth conditions, TSH assay used and timing of TSH determination.^4^ ^31^ Future studies should assess the neurodevelopmental impact of mild iodine deficiency using a prospective design and measuring urinary iodine excretion in the mothers as well as thyroid parameters at different stages of pregnancy starting, if possible, before the conception. Many studies started from the first trimester and disabilities were, most of the times, found when MID appeared before mid-gestation. In addition, neurodevelopmental performance should be assessed at different stages of development, starting earlier than 4–5 years.

The use of neonatal TSH at birth for monitoring iodine status has been questioned. Indeed, several studies have shown that percentage of TSH >5 mIU/L collected after birth has failed to detect mild iodine deficiency.^32–34^ Further studies are needed to assess the association between maternal urinary iodine and neonatal TSH levels in order to define a relevant cut-off point in TSH concentration to define mild iodine deficiency.

In conclusion, the present study found no association between neonatal TSH levels within the range of 0.45–15 mIU/L and psychomotor development of preschool children in Belgium.
